# Brain structure links trait conscientiousness to academic performance

**DOI:** 10.1038/s41598-019-48704-1

**Published:** 2019-08-21

**Authors:** Song Wang, Yajun Zhao, Jingguang Li, Xu Wang, Kui Luo, Qiyong Gong

**Affiliations:** 1 Huaxi MR Research Center (HMRRC), Department of Radiology, West China Hospital of Sichuan University, Chengdu, 610041 China; 2 Department of Psychoradiology, Chengdu Mental Health Center, Chengdu, 610036 China; 3 Psychoradiology Research Unit of Chinese Academy of Medical Sciences (2018RU011), West China Hospital of Sichuan University, Chengdu, 610041 China; 4 School of Sociology and Psychology, Southwest Minzu University, Chengdu, 610041 China; 5 College of Education, Dali University, Dali, 671003 China; 6 School of Life Sciences, Beijing University of Chinese Medicine, Beijing, 100029 China

**Keywords:** Personality, Human behaviour

## Abstract

In the long history of identifying factors to predict academic performance, conscientiousness, a so-called ‘big five’ personality trait describing self-regulation and goal-directed behavior, has emerged as a stable predictor for this purpose. However, the neuroanatomical substrates of trait conscientiousness and the underlying brain mechanism linking trait conscientiousness and academic performance are still largely unknown. Here, we examined these issues in 148 high school students within the same grade by estimating cortical gray matter volume (GMV) utilizing a voxel-based morphometry method based on structural magnetic resonance imaging. A whole-brain regression analysis showed that trait conscientiousness was positively associated with the GMV in the bilateral superior parietal lobe (SPL) and was negatively associated with the GMV in the right middle frontal gyrus (MFG). Furthermore, mediation analysis revealed that trait conscientiousness mediated the influences of the SPL and MFG volume on academic performance. Importantly, our results persisted even when we adjusted for general intelligence, family socioeconomic status and ‘big five’ personality traits other than conscientiousness. Altogether, our study suggests that the GMV in the frontoparietal network is a neurostructural marker of adolescents’ conscientiousness and reveals a potential brain-personality-achievement pathway for predicting academic performance in which gray matter structures affect academic performance through trait conscientiousness.

## Introduction

In the fields of education and psychology, there is a long history of exploring the predictors of academic performance^1^, which is generally assessed using grade point average (GPA) or standardized examinations (e.g., the Stanford Achievement Test and the Achievement College Test)^2,3^. It is well established that a wealth of psychosocial factors, such as family background (e.g., socioeconomic status, SES), general cognitive ability (e.g., intelligence level), motivation and personality traits, are reliable predictors of academic performance^4–10^. Conscientiousness, which is one’s tendency to be goal directed, disciplined, organized and achievement focused, is an aspect of the well-known ‘big five’ personality model^11^ and has been repeatedly shown to be related to academic performance^5,6,8^. For example, a meta-analysis based on 138 samples from 70,926 participants has revealed that trait conscientiousness is moderately associated with students’ academic performance and that this association is independent of general intelligence^5^. Moreover, a recent systematic review of 38 meta-analyses found that among the 16 personality variables related to academic performance, trait conscientiousness showed the largest absolute effect size in the prediction of academic performance^8^. Furthermore, evidence from many longitudinal studies has suggested that trait conscientiousness plays a predictive and causal role in academic performance^12–14^. Here, we employed structural magnetic resonance imaging (sMRI) to examine the neuroanatomical bases of trait conscientiousness and then explored the underlying brain mechanism linking trait conscientiousness and academic performance. We adopted a multidimensional approach (i.e., a brain-personality-achievement approach) to explore the relationships between brain structure, trait conscientiousness and academic performance in a group of high school students (N = 148).

With the rapid development of personality neuroscience in the past decade, an increasing number of neuroimaging studies have been conducted to uncover how trait conscientiousness may be related to individual differences in brain structure^15,16^. However, the neuroanatomical bases of trait conscientiousness in youth populations (e.g., adolescents and children) are still largely unknown. Several sMRI studies in adults have attempted to identify the neurostructural markers of trait conscientiousness but have yielded inconsistent and heterogeneous results^17–27^, which may be due to factors such as heterogeneity in the sample characteristics and differences in the statistical and methodological models used^24,25,28^. For instance, higher trait conscientiousness has been associated with greater gray matter volume (GMV) in the middle frontal gyrus (MFG)^18^, inferior frontal gyrus^21^, orbitofrontal cortex^19^, parieto-occipital sulcus (only in male participants)^25^, superior/inferior frontal gyrus, precuneus, postcentral gyrus, hippocampus, lingual gyrus and caudate nucleus^20^. Higher trait conscientiousness has also been found to be linked to smaller GMV in the fusiform gyrus^18^ and temporal and parietal cortices^20^. Moreover, it has been reported that higher trait conscientiousness is related to increased cortical thickness (CT) in the MFG, orbitofrontal cortex, precuneus, cingulate gyrus, fusiform gyrus and parahippocampal gyrus^22,27^ and to decreased cortical surface area (CSA) in the temporoparietal junction, inferior/middle temporal gyrus and lateral occipital gyrus^17,27^. Furthermore, some studies have reported null results for the association between trait conscientiousness and measures of brain structure (e.g., GMV, CT and CSA)^23,24,26^. To our knowledge, only one recent longitudinal study examined the structural correlates of trait conscientiousness in adolescents (aged 8–19 years), and this research found that trait conscientiousness was associated with the annual percentage change in CT in the frontal and parietal cortices^29^. Considering that adolescence is a period characterized by cognitive and affective changes related to the reorganization of brain structure and function^30,31^, it is necessary to examine the neuroanatomical substrates of trait conscientiousness in adolescents, as the findings observed in adults may not apply to adolescents. Given that no studies have investigated the association between trait conscientiousness and GMV in adolescents, the first goal of the present study was to identify the brain regions in which GMV is associated with trait conscientiousness in a sample of healthy adolescent students within a narrow age range, which may offer sufficient statistical power for whole-brain analyses^32^.

Compared with the increasing number of neural studies on trait conscientiousness, relatively few have examined the neurostructural substrates underlying academic performance. Evidence from the limited literature has suggested that individual differences in academic performance are associated with structural variations in several regions dispersed throughout the frontal, parietal, occipital and temporal lobes^32–34^. For example, through a region-of-interest analysis, a voxel-based morphometry (VBM) study reported that the GMV in the frontal and temporal cortices can explain the variance in children’s academic performance as measured by the Woodcock-Johnson III Tests of Achievement^33^. Another VBM study revealed a relationship between academic performance and the GMV in the dorsolateral prefrontal cortex in a group of senior high school students^34^. Additionally, one study based on CT in junior high school students found that increased CT signal in the temporal, parietal and occipital cortices was associated with increased academic performance as assessed by a statewide standardized exam^32^. Furthermore, the structure of the superior longitudinal fasciculus, an association fiber tract connecting the frontal, parietal, occipital and temporal lobes, is linked to educational attainment^35^ and parent-reported academic achievement^36^ in adolescent students. Given these findings and the association of trait conscientiousness with academic performance, the second goal of the present study was to explore whether the brain regions related to trait conscientiousness can predict academic performance and then to examine the mediation relationship among brain structure, trait conscientiousness and academic performance.

To achieve these goals, we conducted sMRI scans on participants, evaluated their real-world academic performance and administered a standard measure of trait conscientiousness. Here, cortical GMV was estimated using the VBM approach^37^, which is a well-validated and widely used method for investigating the structural features of the brain that underlie personality traits^16,38^. Specifically, the newest version of VBM in Statistical Parametric Mapping software (version: SPM12)^39^ was used to preprocess the image data, given the influences of preprocessing methods on the results of neurostructural investigations^40^. As a comprehensive measure based on CT and CSA, GMV as measured by VBM may reflect the sizes and numbers of unmyelinated neurons and glial cells, along with the volume of the synapses^41,42^. First, a whole-brain regression analysis was performed to identify the brain areas related to trait conscientiousness. Second, correlation analyses and mediation analyses were conducted to probe the associations between trait conscientiousness, academic performance and brain structures. Finally, to assess the specificity of the findings, we carried out supplemental analyses in which several confounding factors (i.e., general intelligence, family SES and ‘big five’ personality traits other than conscientiousness) were controlled for.

## Methods

### Participants

In total, 150 graduates who had recently completed the 12th grade at several local public high schools participated in the present study, which is a part of our ongoing project to explore the neurobiological basis of adolescents’ self-regulation, academic achievement and well-being in Chengdu, China^34,43,44^. Two participants were excluded because of incidental MRI findings (i.e., unusual cysts). Thus, 148 participants (60 females and 88 males) were included in our data analyses. The participants’ ages ranged between 17 and 20 years old (mean age = 18.51 ± 0.55). Through screening with a questionnaire, we limited the subject pool to participants who were right-handed and reported no history of neurological or psychological disorders. It is worth noting that statistical power is extremely important in the field of personality neuroscience, with a total sample size of 150 participants recommended for investigating the neurobiological basis of personality traits^45^. Thus, the sample size of the present study may be able to ensure adequate statistical power. The local research ethics committee of West China Hospital of Sichuan University approved the current study. All experiments were conducted in accordance with the approved guidelines and regulations. Written informed consent was obtained from all participants and their parents prior to our experiments, which were conducted from June 2015 to September 2015.

### Behavioral tests

#### NEO Five-Factor Inventory (NEO-FFI)

The individual differences in trait conscientiousness were assessed using the conscientiousness subscale of the NEO-FFI, which is a popular measure for ‘big five’ personality traits^11^. As a test for the specificity of our results, the other NEO-FFI subscales (i.e., extraversion, neuroticism, openness and agreeableness) were also administered to the participants. The NEO-FFI is a 5-point Likert-type self-report questionnaire that includes 60 items, with 12 items for each subscale. This inventory has been repeatedly used in different Chinese populations and has been shown to have satisfactory validity and reliability^46–48^. In this study, the internal consistency (i.e., Cronbach’s α) of each subscale of the NEO-FFI was adequate: conscientiousness (α = 0.79), extraversion (α = 0.80), neuroticism (α = 0.81), openness (α = 0.74) and agreeableness (α = 0.72).

#### Chinese National University Entrance Exam (CNUEE)

The participants’ academic performance was evaluated using their CNUEE scores^49,50^, which were collected from the databases of the high schools the students attended. The CNUEE is a standardized and scaled measure that consists of six curriculum subjects (i.e., Chinese, Mathematics, English, Physics, Chemistry and Biology). CNUEE scores range between 0 and 750, allowing comparison among students in the same grade and providing the sole criterion for admission to Chinese universities^50,51^. All participants took the CNUEE in June 2015.

#### Raven’s Advanced Progressive Matrices (RAPM)

The 36-item RAPM test^52^, a measure of abstract reasoning ability, was included in the current study to investigate whether general intelligence could affect the associations between trait conscientiousness, gray matter structure and academic performance. For a given item, participants were presented with a picture matrix that was missing one part and were required to select the missing part among 8 options. Participants were required to complete this pencil-and-paper test within 30 minutes^43,53^. RAPM scores were derived based on the number of correct answers, with higher scores suggesting higher levels of general intelligence. In this experiment, the internal consistency of the RAPM was acceptable (α = 0.80).

#### Socioeconomic Status Scale (SSS)

To exclude the potential effects of family SES on the relations among trait conscientiousness, gray matter structure and academic performance, we employed the SSS, which is a single-item scale presenting participants with a drawing of a 10-rung ladder^54^. This scale reflects participants’ perceptions of their parents’ socioeconomic status, including three aspects: education, occupational prestige and income^54^. During the testing, each participant was instructed to indicate the overall level of his/her parents’ socioeconomic status, ranging from 1 (bottom rung) to 10 (top rung). Increasing evidence has revealed that compared to objective measures, SSS is a superior predictor of health-linked outcomes^55^. This scale has been widely used in Chinese populations^56,57^.

### Image acquisition and preprocessing

#### Image acquisition

The sMRI scans were performed with a 3.0 T Siemens-Trio Erlangen scanner (Germany) with a 12-channel head coil. Using a whole-head magnetization-prepared rapid gradient-echo sequence, each participant underwent a T1-weighted structural scan with the following parameters: inversion time = 900 ms, repetition time = 1900 ms, echo time = 2.26 ms, slice thickness = 1 mm, flip angle = 9°, matrix size = 256 × 256, 1 mm isotropic resolution and 176 slices.

#### Image preprocessing

Preprocessing of images was conducted with SPM12^39^. All of the images were first displayed in SPM12 to check for gross anatomical abnormalities or artifacts, and two participants were excluded due to abnormal brain structure. For a more accurate registration, each image was manually reoriented, set to the anterior commissure, and then segmented into three tissue groups (i.e., gray matter, white matter and cerebrospinal fluid) by employing the new segmentation tool in SPM12. Afterwards, registration, normalization and modulation analyses were conducted with DARTEL (diffeomorphic anatomical registration through exponentiated lie) algebra^58^ in SPM12. The gray matter data were aligned, resampled to 2 × 2 × 2 mm^3^, and then transformed to Montreal Neurological Institute (MNI) space. The inverse Jacobian of the local transformations was used to modulate the segmented gray matter data, which allows the volume measurements to be preserved. Subsequently, the normalized and modulated gray matter data were smoothed with an 8-mm full width at half maximum Gaussian kernel. Finally, the resulting data were masked with an absolute threshold masking of 0.2 to remove edge effects around the borders between gray matter and white matter^34,43,59^. In particular, to ensure that there were no crucial anomalies in GMV estimation, a medical radiologist who was blinded to the current study performed visual quality control of each participant’s images in each step. No participants were excluded in this process.

### Statistical analyses

#### GMV-behavior correlation analysis

To identify the brain regions for which GMV is linked with trait conscientiousness, we conducted a whole-brain regression analysis using NEO-FFI conscientiousness scores as the independent variable; voxelwise GMV as the dependent variable; and gender, age and total GMV as the control variables. Additionally, to investigate gender differences in the relations between trait conscientiousness and gray matter structure, we conducted a condition-by-covariate interaction analysis^43,59,60^ using gender as a condition, NEO-FFI conscientiousness scores as the variable of interest and age and total GMV as the variables of no interest. Nonstationary cluster correction was employed to infer the regions of significance, as previous studies have demonstrated that the VBM data are nonstationary (e.g., not uniformly smooth)^37,61^. Specifically, the results were corrected with a threshold (cluster level: *p* < 0.05; voxel level: *p* < 0.0025; nonstationary cluster correction) that has been successfully applied to VBM data^34,42,62^. The above analyses were performed with SPM12.

#### Prediction analysis

To validate the predictability of trait conscientiousness from brain structures, we implemented a balanced fourfold cross-validation procedure utilizing a machine learning method^34,43,44,57,63,64^. For the analysis, a linear regression algorithm was performed using NEO-FFI conscientiousness scores as the dependent variable and the GMV of the brain region as the independent variable. To evaluate how well the dependent variable could be predicted by the independent variable, we calculated *r*_(predicted, observed)_ using a balanced fourfold cross-validation method^34,43,44,57,63,64^. The data were first divided into four subsets to guarantee that there were no significant differences among the distributions of these variables across subsets. Then, the data from three subsets were used to build a linear regression model, with one subset left out. This model was further employed to predict the unused data subset. The value *r*_(predicted, observed)_, which represents the correlation of the actual observed data and the predicted data, was finally obtained after the data of all subsets had been predicted. The significance of *r*_(predicted, observed)_ was determined using a nonparametric testing method^34,43,44,57,63,64^. By randomly shuffling the data of the independent variable, we used the original dependent variable and the shuffled independent variable to compute the *r*_n(predicted, observed)_. The null distribution of *r*_(predicted, observed)_ was obtained by repeating this procedure 5000 times. By subtracting the percentile of the true *r*_(predicted, observed)_ in the null distribution from one, we obtained the significance of *r*_(predicted, observed)_.

#### Mediation analysis

To evaluate the indirect effect of brain structure on academic performance through trait conscientiousness, we conducted a mediation analysis with the SPSS macro PROCESS, including a bootstrapping approach^65^. For this analysis, the GMV of the brain region was considered the predictor variable (X), NEO-FFI conscientiousness scores were considered the mediator variable (M), and CNUEE scores were considered the outcome variable (Y). The indirect effect, referring to the product of path a (i.e., the relation between X and M) and path b (i.e., the relation between M and Y after adjusting for X), was estimated. The point estimates of the indirect effects were considered significant if the bootstrapped 95% confidence intervals (CIs) (5000 iterations) did not include zero.

## Results

### Neurostructural correlates of trait conscientiousness

Table 1 displays the descriptive statistics (i.e., mean, standard deviation, range, skewness and kurtosis) for each behavioral variable included in the present study. The kurtosis and skewness values for all variables ranged between −2 and 2, indicating that the data fulfilled the normality assumption^66,67^. Importantly, trait conscientiousness had no significant relation with age (*r* = −0.02, *p* = 0.788), gender [*t* (146) = 1.92, *p* = 0.057] or total GMV (*r* = 0.12, *p* = 0.138). After controlling for gender and age, the association between trait conscientiousness and total GMV was still not significant (partial *r* = 0.02, *p* = 0.788). We then investigated the structural substrates of trait conscientiousness.

**Table 1:** Descriptive statistics for behavioral measures (N = 148).

Variable	Mean	SD	Range	Skewness	Kurtosis
Age	18.51	0.55	17–20	0.81	1.63
Conscientiousness	39.71	5.73	28–54	0.24	−0.26
Neuroticism	33.27	6.90	15–48	−0.15	−0.37
Extraversion	42.46	6.17	26–58	−0.03	−0.16
Openness	41.39	4.77	31–57	0.38	0.27
Agreeableness	42.95	4.62	31–57	0.11	0.21
General intelligence	25.49	5.12	12–36	−0.22	−0.22
Family SES	5.31	1.45	1–9	−0.30	−0.17
Academic performance	540.25	68.97	317–644	−0.60	−0.04

*Note:* N = number; SD = standard deviation; SES = socioeconomic status.

**Figure 1:**
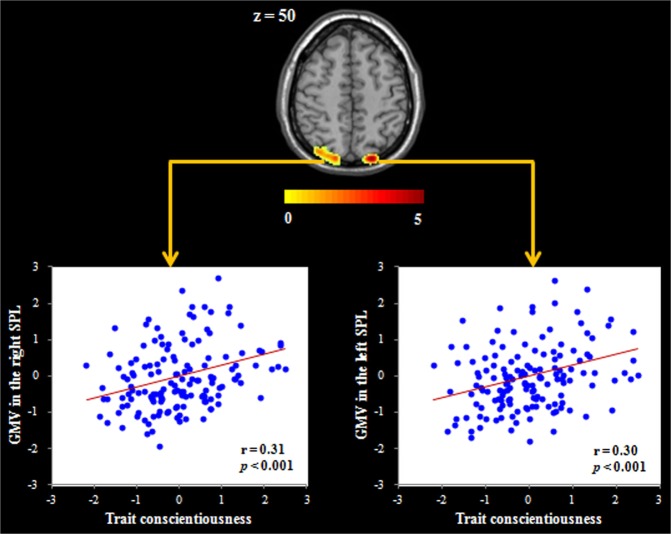
Regional gray matter volume (GMV) related to trait conscientiousness. Brain image showing that the superior parietal lobe (SPL) volume was positively correlated with trait conscientiousness. Scatter plots depicting the correlation between trait conscientiousness and left SPL volume (*r* = 0.30, *p* < 0.001) and right SPL volume (*r* = 0.31, *p* < 0.001). Gender, age and total GMV were regressed out in the analyses.

**Figure 2:**
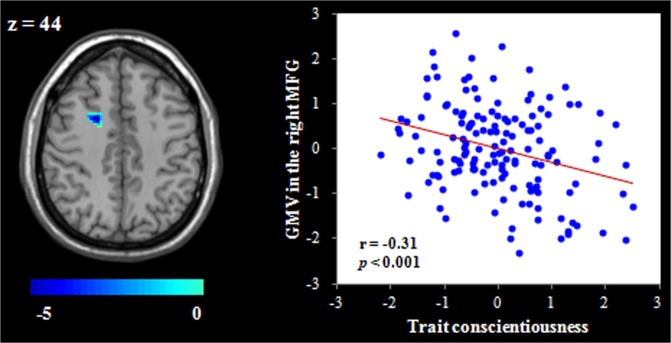
Regional gray matter volume (GMV) related to trait conscientiousness. Brain image showing that the right middle frontal gyrus (MFG) volume was negatively correlated with trait conscientiousness. Scatter plots depicting the correlation between trait conscientiousness and right MFG volume (*r* = −0.31, *p* < 0.001). Gender, age and total GMV were regressed out in the analyses.

The whole-brain regression analysis revealed that after nonstationary cluster correction for multiple testing, trait conscientiousness showed a positive association with the GMV in the bilateral superior parietal lobe (SPL; see Table 2 and Fig. 1). Additionally, trait conscientiousness had a negative association with the GMV in the right MFG (see Table 2 and Fig. 2). No other significant clusters were obtained in this analysis. Given that a previous study has shown gender differences in the association between trait conscientiousness and regional GMV^25^, a condition-by-covariate interaction analysis was performed. The results indicated that there were no gender differences in the association between trait conscientiousness and regional GMV after correcting for multiple comparisons.

**Table 2:** Brain regions whose gray matter volume is correlated with trait conscientiousness.

Region	Peak MNI coordinate			Peak T score	Cluster size (mm^3^)
	x	y	z		
**Positive correlation**					
Left superior parietal lobe	−22	−80	50	4.99	1640
Right superior parietal lobe	36	−78	48	4.84	2664
**Negative correlation**					
Right middle frontal gyrus	20	6	44	−5.18	1936

*Note:* The non-stationary cluster correction was employed to determine the regions of significance with the following settings: *p* < 0.05 at the cluster level and *p* < 0.0025 at the underlying voxel level. MNI, Montreal Neurological Institute.

We next implemented prediction analyses to check the robustness of the relation between trait conscientiousness and the GMV in the brain regions identified from the whole-brain regression analysis. The results showed that trait conscientiousness could be stably predicted by the GMV in the bilateral SPL [for the left SPL: *r*_(predicted, observed)_ = 0.26, *p* < 0.001; for the right SPL: *r*_(predicted, observed)_ = 0.27, *p* < 0.001] and right MFG [*r*_(predicted, observed)_ = −0.27, *p* < 0.001] after controlling for gender, age and total GMV. Given the high correlation between the left SPL and right SPL (*r* = 0.72, *p* < 0.001) and the similar results found in these two regions, we subsequently condensed our analyses by using only the bilateral average SPL volume.

### Brain regions linking trait conscientiousness and academic performance

After assessing the structural substrates of trait conscientiousness, we further investigated the underlying brain-trait conscientiousness mechanism in predicting academic performance by collecting CNUEE scores. First, we verified the positive correlation of trait conscientiousness with academic performance (*r* = 0.30, *p* < 0.001). Further regression analysis revealed that trait conscientiousness explained additional variance in academic performance after adjusting for gender, age and total GMV (*△R*^2^ = 7.4%, *β* = 0.28, *p* < 0.001). Second, we tested whether academic performance could be predicted by the GMV in the identified brain regions. We found a significant correlation between bilateral SPL volume and academic performance (*r* = 0.24, *p* = 0.003). However, we did not find a significant correlation between right MFG volume and academic performance (*r* = −0.08, *p* = 0.346). Further regression analysis revealed that the bilateral SPL volume (*△R*^2^ = 3.9%, *β* = 0.20, *p* = 0.012) but not the right MFG volume (*△R*^2^ = 0.8%, *β* = −0.09, *p* = 0.278) explained additional variance in academic performance after adjusting for gender, age and total GMV.

**Figure 3:**
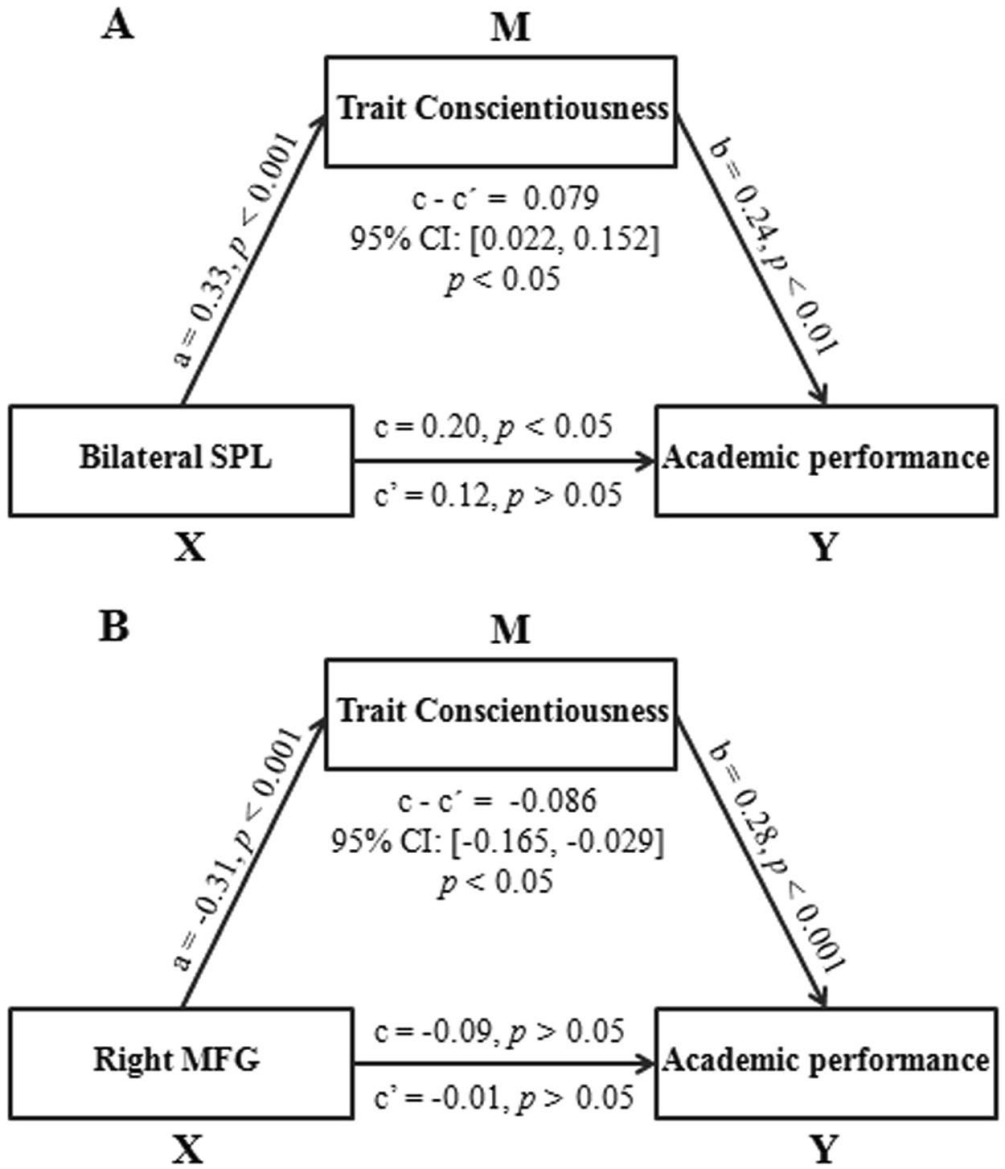
Trait conscientiousness mediates the influence of the bilateral SPL (**A**) and right MFG (**B**) volume on academic performance. Standardized regression coefficients were presented in the path diagrams. Gender, age and total gray matter volume were regressed out in the models. SPL, superior parietal lobe; MFG, middle frontal gyrus.

We then carried out mediation analyses to test whether trait conscientiousness could mediate the link between regional GMV and academic performance. Interestingly, trait conscientiousness played a mediating role in the relationship between bilateral SPL volume and academic performance (indirect effect = 0.088, 95% CI = [0.030, 0.166], *p* < 0.05). Given that many studies have suggested that direct effects are not an appropriate premise for testing indirect effects^68^, we also investigated the mediating role of trait conscientiousness in the association between right MFG volume and academic performance. The results showed that trait conscientiousness can also mediate the relationship between right MFG volume and academic performance (indirect effect = −0.092, 95% CI = [−0.177, −0.036], *p* < 0.05). Even after controlling for gender, age and total GMV, trait conscientiousness can still mediate the influences of the GMV in the bilateral SPL (indirect effect = 0.079, 95% CI = [0.022, 0.152], *p* < 0.05; Fig. 3A) and right MFG (indirect effect = −0.086, 95% CI = [−0.165, −0.029], *p* < 0.05; Fig. 3B) on academic performance.

Furthermore, we performed several other mediation analyses to explore the directionality of the relations between regional GMV, trait conscientiousness and academic performance. In particular, given that several previous longitudinal studies have suggested that trait conscientiousness is a stable antecedent for academic performance^12–14^, there are three possible mediation models for the association between brain structure, trait conscientiousness and academic performance (Model 1: X = SPL or MFG, M = trait conscientiousness, Y = academic performance; Model 2: X = trait conscientiousness, M = SPL or MFG, Y = academic performance; Model 3: X = trait conscientiousness, M = academic performance, Y = SPL or MFG). In addition to Model 1, which was investigated in the above analyses, we further examined Model 2 and Model 3 and found no significant indirect effects for either of these two models. Specifically, the bilateral SPL volume (indirect effect = 0.041, 95% CI = [−0.007, 0.099], *p* > 0.05) or right MFG volume (indirect effect = −0.002, 95% CI = [−0.052, 0.051], *p* > 0.05) could not mediate the effect of trait conscientiousness on academic performance; similarly, academic performance could not mediate the effect of trait conscientiousness on the GMV in the bilateral SPL (indirect effect = 0.034, 95% CI = [−0.002, 0.093], *p* > 0.05) or right MFG (indirect effect = −0.002, 95% CI = [−0.051, 0.044], *p* > 0.05). Gender, age and total GMV were controlled for in these analyses. Therefore, these findings suggest that the GMV in the bilateral SPL and right MFG might affect academic performance through trait conscientiousness.

### Supplemental analyses

To test the specific nature of the above findings, we carried out supplemental analyses to exclude the possible effects of general intelligence, family SES and ‘big five’ personality traits other than conscientiousness (i.e., extraversion, neuroticism, openness, agreeableness). Table 3 lists the correlations of all behavioral measures included in this study.

**Table 3:** Correlations of all measures included in the present study.

	1	2	3	4	5	6	7	8
1. Age	1.00							
2. Conscientiousness	−0.02	1.00						
3. Neuroticism	0.09	−0.40^***^	1.00					
4. Extraversion	−0.02	0.18^*^	−0.36^***^	1.00				
5. Openness	0.03	0.34^***^	−0.21^**^	0.28^***^	1.00			
6. Agreeableness	0.01	0.22^**^	−0.33^***^	0.06	0.19^*^	1.00		
7. General intelligence	−0.06	0.03	−0.02	−0.09	0.11	0.17^*^	1.00	
8. Family SES	−0.01	0.16^*^	−0.11	0.11	0.08	0.03	0.01	1.00
9. Academic performance	0.02	0.30^***^	−0.14	−0.12	0.14	0.13	0.30^***^	0.01

*Note:* SES = socioeconomic status. ^*^p < 0.05; ^**^p < 0.01; ^***^p < 0.001.

First, we checked whether general intelligence could affect our results because converging evidence has shown that general intelligence is stably associated with academic performance^4,10^ and brain structure^69^. Behaviorally, when general intelligence was added as a covariate, trait conscientiousness had incremental predictive ability for academic performance (*△R*^2^ = 7.7%, *β* = 0.28, *p* < 0.001). Neuroanatomically, trait conscientiousness was still correlated with the GMV in the bilateral SPL (partial *r* = 0.33, *p* < 0.001) and right MFG (partial *r* = −0.31, *p* < 0.001), even after controlling for general intelligence, gender, age and total GMV. Importantly, after adjusting for general intelligence, gender, age and total GMV, the mediating effects of trait conscientiousness were still significant: for the relation between bilateral SPL GMV and academic performance, indirect effect = 0.084, 95% CI = [0.030, 0.158], *p* < 0.05; for the relation between right MFG GMV and academic performance, indirect effect = −0.094, 95% CI = [−0.179, −0.038], *p* < 0.05. Thus, our findings are independent of general intelligence.

Second, because SES has been found to be a stable predictor of academic performance and brain structure^9,32,70^, we examined whether our results were influenced by the participants’ family SES. Behaviorally, when family SES was added as a covariate, trait conscientiousness had incremental predictive ability for academic performance (*△R*^2^ = 7.6%, *β* = 0.28, *p* < 0.001). Neuroanatomically, trait conscientiousness was still correlated with the GMV in the bilateral SPL (partial *r* = 0.32, *p* < 0.001) and right MFG (partial *r* = −0.28, *p* < 0.001), even after controlling for family SES, gender, age and total GMV. Importantly, after adjusting for family SES, gender, age and total GMV, the mediating effects of trait conscientiousness were still significant: for the relation between bilateral SPL GMV and academic performance, indirect effect = 0.078, 95% CI = [0.026, 0.153], *p* < 0.05; for the relation between right MFG GMV and academic performance, indirect effect = −0.080, 95% CI = [−0.155, −0.028], *p* < 0.05. In summary, our findings are not affected by family SES.

Third, we evaluated the possible influence of other ‘big five’ personality traits (i.e., extraversion, neuroticism, openness, agreeableness) on the associations between trait conscientiousness, brain structure and academic performance. Behaviorally, when the rest of the ‘big five’ personality traits were included as covariates, trait conscientiousness had incremental predictive ability for academic performance (*△R*^2^ = 4.7%, *β* = 0.25, *p* = 0.006). Neuroanatomically, trait conscientiousness was still correlated with the GMV in the bilateral SPL (partial *r* = 0.31, *p* < 0.001) and right MFG (partial *r* = −0.27, *p* < 0.001), even after controlling for other ‘big five’ personality traits, gender, age and total GMV. Importantly, after adjusting for other ‘big five’ personality traits, gender, age and total GMV, the mediating effects of trait conscientiousness were still significant: for the relation between bilateral SPL GMV and academic performance, indirect effect = 0.060, 95% CI = [0.011, 0.132], *p* < 0.05; for the relation between right MFG GMV and academic performance, indirect effect = −0.062, 95% CI = [−0.134, −0.018], *p* < 0.05. Therefore, our results show some degree of specificity, although other ‘big five’ personality traits reduced the effect sizes of the results.

## Discussion

The present research was conducted to investigate the structural brain substrates underlying adolescent conscientiousness and to explore the nature of the relationships among gray matter structure, trait conscientiousness and academic performance. We observed that the GMV in the bilateral SPL was positively linked to trait conscientiousness, which is in line with two previous VBM studies reporting gray matter variations in the SPL and individual differences in trait conscientiousness^18,20^. Furthermore, our result is consistent with the finding of a recent longitudinal experiment showing an association between annual percentage change in CT in the SPL and trait conscientiousness in adolescents^29^. The functioning of the SPL has also been found to be associated with trait conscientiousness^71–73^. For example, evidence from an electroencephalography study showed a positive relation between trait conscientiousness and photic driving in the beta frequency band (i.e., the mean of 13, 16 and 18 Hz stimulation frequency) in the parietal lobe^73^. In addition, through an independent component analysis based on resting-state functional MRI (fMRI), Sampaio *et al*. (2014) reported a positive association between trait conscientiousness and spontaneous activity in the SPL^72^. Furthermore, a task-based fMRI investigation found that during a 3-back working memory task, trait conscientiousness was related to the functional connectivity from the SPL to the MFG^71^. The SPL is generally considered a core brain region in the multiple-demand system^74^, which is hypothesized to be crucial for conscientious tendencies^27^. In summary, our finding regarding the relationship between trait conscientiousness and SPL volume may substantiate the role of SPL structure and function in trait conscientiousness.

Moreover, trait conscientiousness showed a negative association with the GMV in the right MFG. This finding fits well with several previous sMRI investigations that have demonstrated associations between trait conscientiousness and measures of brain structure (e.g., GMV and CSA) in MFG^18,19,27^. Evidence from lesion research has suggested that focal damage to the MFG leads to reduced levels of executive function and conscientiousness, which presents direct evidence for the causal role of MFG in trait conscientiousness^75^. Moreover, it has been reported that MFG activity during resting-state fMRI scans can predict individual differences in trait conscientiousness^76^. Broadly, the MFG is identified as crucial for behaviors related to self-regulation behaviors^77^, which have self-evident relevance to trait conscientiousness^78^. Evidence from several task-based fMRI studies has suggested that individual differences in trait conscientiousness are linked to the activity and connectivity in the MFG during self-regulation-related tasks such as delay discounting^79^, inhibitory control^80^ and working memory^71^. Additionally, the negative relationship between trait conscientiousness and MFG volume may reflect the processing of synaptic pruning and myelination during development in some brain regions, which may lead to improved efficiency in certain cognitive and noncognitive capacities^31,81,82^. For example, decreased MFG volume has been linked to increases in high-order variables such as quality of life^41^, social well-being^83^ and creative cognitive ability^84^. Therefore, synaptic pruning and myelination in the MFG during development may increase efficiency in self-regulation-related capacities (e.g., executive function, inhibitory control and delay discounting), which may increase conscientious tendencies.

Interestingly, we found that trait conscientiousness served as a mediator in the link between SPL volume and academic performance. Behaviorally, the association of trait conscientiousness with academic performance has been well established in previous investigations^5,6,8,12–14^. This association was replicated in the current sample (*r* = 0.30, p < 0.001). Hierarchical regression analysis further revealed that even after we excluded the influences of general intelligence, family SES, and ‘big five’ personality traits other than conscientiousness, as well as gender, age and total GMV, trait conscientiousness still accounted for additional variance in academic performance (*R*^2^ = 5.5%, *β* = 0.27, *p* < 0.001). Therefore, our study may present further evidence for the predictive role of trait conscientiousness in academic performance. Neuroanatomically, we observed that the variance in academic performance could be explained by the GMV in the SPL. Although there are currently no reports of an association between SPL structure and academic performance, there is some evidence suggesting a role of SPL function in measures of academic performance^85,86^. For example, SPL activity during a memory task has been correlated with educational attainment in a group of young adults^86^. One recent study further revealed that the SPL activity of adolescent students during working memory tasks can predict variance in academic performance on the mathematics section of the Massachusetts Comprehensive Assessment System^85^. In general, the SPL is associated with multiple high-level psychological functions, including top-down and goal-directed attention^87^, working memory^88^, executive function^89^, spatial ability^90^ and problem solving related to planning and visuospatial reasoning^91^. Thus, the volume of the SPL may affect academic performance through these psychological attributes, which are regarded as important predictors of academic performance^6,92–95^. Altogether, our study indicates that trait conscientiousness may be a potential mechanism linking the GMV in the SPL to academic performance.

Additionally, we found that trait conscientiousness mediated the effects of the GMV in the right MFG on academic performance, although there was no significant association between right MFG volume and academic performance. This finding suggests that experimentally modulating right MFG structure and function, such as with transcranial direct current stimulation^96^ and neurofeedback training^97^, may be a promising approach to promote conscientiousness in adolescent students, which might, in turn, enhance academic performance. Notably, further longitudinal or experimental evidence is needed to verify the directionality of the association in this process. Moreover, although no significant association between MFG volume and academic performance was observed in the present study, there is room for future studies exploring this association because some empirical evidence has shown a crucial role of MFG structure and function in academic performance^33,34,36,86,98^. In summary, our finding may present an underlying pathway in which the GMV in the right MFG indirectly affects academic performance by modulating trait conscientiousness.

Our research has several limitations that should be acknowledged. First, we used only a self-reported NEO-FFI questionnaire to assess trait conscientiousness, although the validity and reliability of this measure have been well established^11,46–48^. It is necessary for future studies to use multiple techniques (e.g., peer rating or experience sampling) to decrease the response bias and enhance measurement accuracy. Additionally, the measurement of academic performance relied on a single university entrance exam (i.e., CNUEE), although it is a well-standardized and authoritative test for assessing academic performance in Chinese high school students^49–51^. There is some evidence to suggest that in other countries, the university entrance exam may be heavily loaded to general intelligence and behave differently from measures such as GPA^2,6^. Thus, future studies will need to use other measures of academic performance (e.g., GPA) to validate our findings. Second, in our mediation analyses, we tested three possible mediation models, given that several previous longitudinal studies have suggested that trait conscientiousness is a stable predictor of academic performance^12–14^, and we found only one significant model. However, due to the correlational nature of the present study, we could not exclude other possible mediation associations in which academic performance is an antecedent for trait conscientiousness, which reflects hypotheses linking to cognitive development or self-perception. For example, in our dataset, academic performance could mediate the impact of bilateral SPL volume on trait conscientiousness (indirect effect = 0.046, 95% CI = [0.013, 0.102], *p* < 0.05). Thus, it is difficult to draw a causal conclusion regarding the relationships between brain structure, trait conscientiousness and academic performance because we based our research on cross-sectional data. Future studies using more sophisticated methods (e.g., experimental or longitudinal designs) are needed to establish the directions of the causal relationships between brain structure, trait conscientiousness and academic performance. Third, we revealed only that the GMV in the SPL and MFG is linked with trait conscientiousness; we failed to observe the relations found in prior investigations between trait conscientiousness and the GMV in other cortical and subcortical regions^17,19,20,22,25,27,29^. Given that we employed only one measure of brain structure (i.e., GMV) and no functional measures, other measures of brain structure (e.g., CT and CSA) and function (e.g., task-based and resting-state brain activity) may be used to further explore the neurobiological bases of trait conscientiousness and their relations to academic performance.

In conclusion, this research provides initial evidence for neurostructural markers underlying adolescent conscientiousness by revealing that the GMV in the bilateral SPL and right MFG is linked to trait conscientiousness in a sample of high school students. Additionally, our study presents pioneering evidence suggesting that the association between SPL/MFG volume and academic performance is mediated by trait conscientiousness. These findings suggest the important role of trait conscientiousness and the GMV in the frontoparietal network in the prediction of academic performance, and they also introduce new research directions for examining how brain measures affect academic performance through individual personality traits. Finally, our findings may add to the development of psychoradiology, a new field of radiology with the purpose of not only improving our understanding of the mechanisms underlying psychiatric disorders, but also have great potential to play the clinical role in guiding diagnostic and treatment planning decisions in psychiatric patients^99–102^.

## Data Availability

The data and code that support the findings of this study are available from the corresponding author upon reasonable request. And the data and code sharing adopted by the authors comply with the requirements of the funding institute, and comply with institutional ethics approval.

## References

[1] Lavin, D. E. The prediction of academic performance: A theoretical analysis and review of research. *Russell Sage Foundation, New York* (1965).

[2] Fan XT, Chen M (2001). Parental involvement and students’ academic achievement: A meta-analysis. Educ Psychol Rev.

[3] Seipp B (1991). Anxiety and academic performance: A meta-analysis of findings. Anxiety Research.

[4] Deary IJ, Strand S, Smith P, Fernandes C (2007). Intelligence and educational achievement. Intelligence.

[5] Poropat AE (2009). A meta-analysis of the five-factor model of personality and academic performance. Psychological Bulletin.

[6] Richardson M, Abraham C, Bond R (2012). Psychological correlates of university students’ academic performance: A systematic review and meta-analysis. Psychological Bulletin.

[7] Robbins SB (2004). Do psychosocial and study skill, factors predict college outcomes? A meta-analysis. Psychological Bulletin.

[8] Schneider M, Preckel F (2017). Variables associated with achievement in higher education: A systematic review of meta-analyses. Psychological Bulletin.

[9] Sirin SR (2005). Socioeconomic status and academic achievement: A meta-analytic review of research. Rev Educ Res.

[10] Sternberg RJ, Grigorenko EL, Bundy DA (2001). The predictive value of IQ. Merrill Palmer Quart.

[11] Costa PT, McCrae RR (1992). Normal personality assessment in clinical practice: The NEO Personality Inventory. Psychol Assessment.

[12] Chamorro-Premuzic T, Furnham A (2003). Personality predicts academic performance: Evidence from two longitudinal university samples. J Res Pers.

[13] Heaven PCL, Ciarrochi J (2008). Parental styles, conscientiousness, and academic performance in high school: A three-wave longitudinal study. Pers Soc Psychol B.

[14] Heaven PCL, Clarrochi J, Vialle W (2007). Conscientiousness and Eysenckian psychoticism as predictors of school grades: A one-year longitudinal study. Pers Indiv Differ.

[15] Abram SV, DeYoung CG (2017). Using personality neuroscience to study personality disorder. Personal Disord.

[16] Yarkoni Tal (2015). Neurobiological substrates of personality: A critical overview. APA handbook of personality and social psychology, Volume 4: Personality processes and individual differences.

[17] Bjornebekk A (2013). Neuronal correlates of the five factor model (FFM) of human personality: Multimodal imaging in a large healthy sample. NeuroImage.

[18] DeYoung CG (2010). Testing predictions from personality neuroscience: Brain structure and the big five. Psychol Sci.

[19] Jackson J, Balota DA, Head D (2011). Exploring the relationship between personality and regional brain volume in healthy aging. Neurobiol Aging.

[20] Kapogiannis D, Sutin A, Davatzikos C, Costa P, Resnick S (2013). The five factors of personality and regional cortical variability in the Baltimore longitudinal study of aging. Human Brain Mapping.

[21] Chen C, Mao Y, Luo J, He L, Jiang Q (2018). Regional gray matter volume mediates the relationship between conscientiousness and expressive suppression. Front Hum Neurosci.

[22] Lewis GJ (2018). Widespread associations between trait conscientiousness and thickness of brain cortical regions. NeuroImage.

[23] Li T (2017). Neuronal correlates of individual differences in the big five personality traits: Evidences from cortical morphology and functional homogeneity. Front Neurosci.

[24] Liu WY (2013). The Big Five of Personality and structural imaging revisited: a VBM - DARTEL study. Neuroreport.

[25] Nostro AD, Muller VI, Reid AT, Eickhoff SB (2017). Correlations between personality and brain structure: A crucial role of gender. Cereb Cortex.

[26] Privado J, Roman FJ, Saenz-Urturi C, Burgaleta M, Colom R (2017). Gray and white matter correlates of the big five personality traits. Neuroscience.

[27] Riccelli R, Toschi N, Nigro S, Terracciano A, Passamonti L (2017). Surface-based morphometry reveals the neuroanatomical basis of the five-factor model of personality. Social Cognitive and Affective Neuroscience.

[28] Hu XC (2011). Voxel-based morphometry studies of personality: Issue of statistical model specification-effect of nuisance covariates. NeuroImage.

[29] Ferschmann L (2018). Personality traits are associated with cortical development across adolescence: A longitudinal structural MRI study. Child Dev.

[30] Foulkes L, Blakemore SJ (2018). Studying individual differences in human adolescent brain development. Nature Neuroscience.

[31] Konrad K, Firk C, Uhlhaas PJ (2013). Brain development during adolescence: Neuroscientific insights into this developmental period. Deutsches Arzteblatt International.

[32] Mackey AP (2015). Neuroanatomical correlates of the income-achievement gap. Psychol Sci.

[33] Hair NL, Hanson JL, Wolfe BL, Pollak SD (2015). Association of child poverty, brain development, and academic achievement. Jama Pediatr.

[34] Wang S (2017). Brain structure linking delay discounting and academic performance. Human Brain Mapping.

[35] Noble KG, Korgaonkar MS, Grieve SM, Brickman AM (2013). Higher education is an age-independent predictor of white matter integrity and cognitive control in late adolescence. Developmental Sci.

[36] Rosen ML, Sheridan MA, Sambrook KA, Meltzoff AN, McLaughlin KA (2018). Socioeconomic disparities in academic achievement: A multi-modal investigation of neural mechanisms in children and adolescents. NeuroImage.

[37] Ashburner J, Friston KJ (2000). Voxel-based morphometry—The methods. NeuroImage.

[38] Lerch JP (2017). Studying neuroanatomy using MRI. Nature Neuroscience.

[39] Friston, K. J., Ashburner, J., Kiebel, S. J., Nichols, T. E. & Penny, W. D. Statistical parametrc mapping: The analysis of functional brain images. London: Academic Press (2007).

[40] Martínez K (2015). Reproducibility of brain-cognition relationships using three cortical surface-based protocols: An exhaustive analysis based on cortical thickness. Human Brain Mapping.

[41] Takeuchi H (2014). Anatomical correlates of quality of life: Evidence from voxel-based morphometry. Human Brain Mapping.

[42] Takeuchi H (2012). A voxel-based morphometry study of gray and white matter correlates of a need for uniqueness. NeuroImage.

[43] Wang S (2018). Neuroanatomical correlates of grit: Growth mindset mediates the association between gray matter structure and trait grit in late adolescence. Human Brain Mapping.

[44] Wang S (2017). Hope and the brain: Trait hope mediates the protective role of medial orbitofrontal cortex spontaneous activity against anxiety. NeuroImage.

[45] Mar RA, Spreng RN, DeYoung CG (2013). How to produce personality neuroscience research with high statistical power and low additional cost. Cogn Affect Behav Ne.

[46] Yang J (1999). Cross-cultural personality assessment in psychiatric populations: The NEO-PI-R in the People’s Republic of China. Psychol Assessment.

[47] Yu XN, Zhang JX (2007). Factor analysis and psychometric evaluation of the Connor-Davidson Resilience Scale (CD-RISC) with Chinese people. Soc Behav Personal.

[48] Zhang LF (2003). Does the big five predict learning approaches?. Pers Indiv Differ.

[49] Bai C, Chi W, Qian X (2014). Do college entrance examination scores predict undergraduate GPAs? A tale of two universities. China Economic Review.

[50] Davey G, De Lian C, Higgins L (2007). The university entrance examination system in China. Journal of further and Higher Education.

[51] Liu H, Wu Q (2006). Consequences of college entrance exams in China and the reform challenges. KEDI. Journal of Educational Policy.

[52] Raven J (2000). The Raven’s Progressive Matrices: Change and stability over culture and time. Cognitive Psychology.

[53] Chen Z, De Beuckelaer A, Wang X, Liu J (2017). Distinct neural substrates of visuospatial and verbal-analytic reasoning as assessed by Raven’s Advanced Progressive Matrices. Sci Rep-Uk.

[54] Adler NE, Epel ES, Castellazzo G, Ickovics JR (2000). Relationship of subjective and objective social status with psychological and physiological functioning: Preliminary data in healthy white women. Health Psychol.

[55] Cundiff JM, Matthews KA (2017). Is subjective social status a unique correlate of physical health? A meta-analysis. Health Psychol.

[56] Hu PF, Adler NE, Goldman N, Weinstein M, Seeman TE (2005). Relationship between subjective social status and measures of health in older Taiwanese persons. J Am Geriatr Soc.

[57] Kong F, Wang X, Hu SY, Liu J (2015). Neural correlates of psychological resilience and their relation to life satisfaction in a sample of healthy young adults. NeuroImage.

[58] Ashburner J (2007). A fast diffeomorphic image registration algorithm. NeuroImage.

[59] Ming D (2016). Examining brain structures associated with the motive to achieve success and the motive to avoid failure: A voxel-based morphometry study. Soc Neurosci-Uk.

[60] Yamasue H (2008). Sex-linked neuroanatomical basis of human altruistic cooperativeness. Cereb Cortex.

[61] Hayasaka S, Phan KL, Liberzon I, Worsley KJ, Nichols TE (2004). Nonstationary cluster-size inference with random field and permutation methods. NeuroImage.

[62] Kong F (2015). Examining gray matter structures associated with individual differences in global life satisfaction in a large sample of young adults. Social Cognitive and Affective Neuroscience.

[63] Supekar K (2013). Neural predictors of individual differences in response to math tutoring in primary-grade school children. Proc Natl Acad Sci USA.

[64] Yang ZT (2016). Neural univariate activity and multivariate pattern in the posterior superior temporal sulcus differentially encode facial expression and identity. Sci Rep-Uk.

[65] Hayes, A. F. Introduction to mediation, moderation, and conditional process analysis: A regression-based approach. *New York, NY: The Guilford Press* (2013).

[66] Chalbot S (2010). Cerebrospinal fluid secretory Ca-dependent phospholipase A2 activity: A biomarker of blood-cerebrospinal fluid barrier permeability. Neurosci Lett.

[67] Lawson J, Baron-Cohen S, Wheelwright S (2004). Empathising and systemising in adults with and without Asperger Syndrome. J Autism Dev Disord.

[68] Zhao XS, Lynch JG, Chen QM (2010). Reconsidering Baron and Kenny: Myths and truths about mediation analysis. J Consum Res.

[69] Basten U, Hilger K, Fiebach CJ (2015). Where smart brains are different: A quantitative meta-analysis of functional and structural brain imaging studies on intelligence. Intelligence.

[70] Noble KG (2015). Family income, parental education and brain structure in children and adolescents. Nature Neuroscience.

[71] Dima D, Friston KJ, Stephan KE, Frangou S (2015). Neuroticism and conscientiousness respectively constrain and facilitate short-term plasticity within the working memory neural network. Human Brain Mapping.

[72] Sampaio A, Soares JM, Coutinho J, Sousa N, Goncalves OF (2014). The big five default brain: Functional evidence. Brain Struct Funct.

[73] Stough C, Donaldson C, Scarlata B, Ciorciari J (2001). Psychophysiological correlates of the NEO PI-R openness, agreeableness and conscientiousness: Preliminary results. Int J Psychophysiol.

[74] Duncan J (2013). The structure of cognition: Attentional episodes in mind and brain. Neuron.

[75] Forbes CE (2014). The role of executive function and the dorsolateral prefrontal cortex in the expression of neuroticism and conscientiousness. Soc Neurosci-Uk.

[76] Kunisato Y (2011). Personality traits and the amplitude of spontaneous low-frequency oscillations during resting state. Neurosci Lett.

[77] Kelley WM, Wagner DD, Heatherton TF (2015). In search of a human self-regulation system. Annu Rev Neurosci.

[78] McCrae, R. R. & Löckenhoff, C. E. Self-regulation and the five-factor model of personality traits. In *Hoyle, R. H. (Ed.), Handbook of personality and self-regulation (pp. 145–168).: Wiley-Blackwell* (2010).

[79] Manning J (2014). Personality influences temporal discounting preferences: Behavioral and brain evidence. NeuroImage.

[80] Rodrigo AH (2016). Linking trait-based phenotypes to prefrontal cortex activation during inhibitory control. Social Cognitive and Affective Neuroscience.

[81] Blakemore SJ (2012). Development of the social brain in adolescence. J Roy Soc Med.

[82] Spear LP (2000). The adolescent brain and age-related behavioral manifestations. Neurosci Biobehav R.

[83] Kong F, Hu SY, Xue S, Song YY, Liu J (2015). Extraversion mediates the relationship between structural variations in the dorsolateral prefrontal cortex and social well-being. NeuroImage.

[84] Chen QL (2018). Longitudinal alterations of frontoparietal and frontotemporal networks predict future creative cognitive ability. Cereb Cortex.

[85] Finn AS (2017). Functional brain organization of working memory in adolescents varies in relation to family income and academic achievement. Developmental Sci.

[86] Springer MV, McIntosh AR, Winocur G, Grady CL (2005). The relation between brain activity during memory tasks and years of education in young and older adults. Neuropsychology.

[87] Behrmann M, Geng JJ, Shomstein S (2004). Parietal cortex and attention. Curr Opin Neurobiol.

[88] Koenigs M, Barbey AK, Postle BR, Grafman J (2009). Superior parietal cortex is critical for the manipulation of information in working memory. J Neurosci.

[89] Niendam TA (2012). Meta-analytic evidence for a superordinate cognitive control network subserving diverse executive functions. Cogn Affect Behav Ne.

[90] Husain M, Nachev P (2007). Space and the parietal cortex. Trends in Cognitive Sciences.

[91] Newman SD, Carpenter PA, Varma S, Just MA (2003). Frontal and parietal participation in problem solving in the Tower of London: fMRI and computational modeling of planning and high-level perception. Neuropsychologia.

[92] Higgins DM, Peterson JB, Pihl RO, Lee AGM (2007). Prefrontal cognitive ability, intelligence, big five personality, and the prediction of advanced academic and workplace performance. J Pers Soc Psychol.

[93] Mayes SD, Calhoun SL, Bixler EO, Zimmerman DN (2009). IQ and neuropsychological predictors of academic achievement. Learn Individ Differ.

[94] Rohde TE, Thompson LA (2007). Predicting academic achievement with cognitive ability. Intelligence.

[95] Steinmayr R, Ziegler M, Trauble B (2010). Do intelligence and sustained attention interact in predicting academic achievement?. Learn Individ Differ.

[96] Salehinejad MA, Nejati V, Derakhshan M (2017). Neural correlates of trait resiliency: Evidence from electrical stimulation of the dorsolateral prefrontal cortex (dLPFC) and orbitofrontal cortex (OFC). Pers Indiv Differ.

[97] Johnston SJ, Boehm SG, Healy D, Goebel R, Linden DEJ (2010). Neurofeedback: A promising tool for the self-regulation of emotion networks. NeuroImage.

[98] Horowitz-Kraus T (2015). Predicting better performance on a college preparedness test from narrative comprehension at the age of 6 years: An fMRI study. Brain research.

[99] Kressel Herbert Y. (2017). Setting Sail: 2017. Radiology.

[100] Lui, S., Zhou, X. J., Sweeney, J. A. & Gong, Q. Psychoradiology: The frontier of neuroimaging in psychiatry. *Radiology* **281**, 357–372 (2016). 10.1148/radiol.2016152149 PMC5084981 27755933

[101] Port John D. (2018). Diagnosis of Attention Deficit Hyperactivity Disorder by Using MR Imaging and Radiomics: A Potential Tool for Clinicians. Radiology.

[102] Sun, H. *et al.* Psychoradiologic utility of MR imaging for diagnosis of attention deficit hyperactivity disorder: A radiomics analysis. *Radiology* **287**, 620–630 (2017). 10.1148/radiol.2017170226 29165048

